# Monitor units are not predictive of neutron dose for high-energy IMRT

**DOI:** 10.1186/1748-717X-7-138

**Published:** 2012-08-10

**Authors:** Roger A Hälg, Jürgen Besserer, Markus Boschung, Sabine Mayer, Uwe Schneider

**Affiliations:** 1 Institute for Radiotherapy, Radiotherapie Hirslanden AG, Aarau, Switzerland; 2 Vetsuisse Faculty, University of Zurich, Zurich, Switzerland; 3 Division for Radiation Safety and Security, Paul Scherrer Institut, Villigen, Switzerland

## Abstract

**Background:**

Due to the substantial increase in beam-on time of high energy intensity-modulated radiotherapy (>10 MV) techniques to deliver the same target dose compared to conventional treatment techniques, an increased dose of scatter radiation, including neutrons, is delivered to the patient. As a consequence, an increase in second malignancies may be expected in the future with the application of intensity-modulated radiotherapy. It is commonly assumed that the neutron dose equivalent scales with the number of monitor units.

**Methods:**

Measurements of neutron dose equivalent were performed for an open and an intensity-modulated field at four positions: inside and outside of the treatment field at 0.2 cm and 15 cm depth, respectively.

**Results:**

It was shown that the neutron dose equivalent, which a patient receives during an intensity-modulated radiotherapy treatment, does not scale with the ratio of applied monitor units relative to an open field irradiation. Outside the treatment volume at larger depth 35% less neutron dose equivalent is delivered than expected.

**Conclusions:**

The predicted increase of second cancer induction rates from intensity-modulated treatment techniques can be overestimated when the neutron dose is simply scaled with monitor units.

## Background

With the application of new radiation treatment modalities such as intensity-modulated radiotherapy (IMRT) or intensity-modulated arc-therapy, increased tumor control probabilities are anticipated. However, with the application of these treatment techniques also a larger number of secondary cancers is expected. Some scientists believe that we will see an increase in second malignancies due to the substantial increase in beam-on time of IMRT techniques to deliver the same target dose compared to conventional treatment techniques [1,2]. A consequence of the extended beam-on time is an increased dose of scatter radiation, including neutrons, which affects the whole patient. The neutrons could lead to a considerable contribution to the integral dose, in particular, since neutrons have a large quality factor and thus even a small physical dose can result in substantial biological effects.

Most measurements and estimates of neutron dose equivalent from radiotherapy treatments found in literature are usually given in operational dose quantities, which represent effective dose and thus a person risk. A compilation of current literature is listed in the review article by Xu et al. [3]. It should be noted that the neutron dose is only one contribution to integral dose and person risk. Others are for example dose contributions from scattered photons, leakage radiation, products from inelastic nuclear reactions and imaging modalities.

In this report neutron detectors were used, which were calibrated to measure local neutron dose equivalent, at different depths in a solid water phantom in- and outside of the primary radiation field for an open and intensity-modulated radiation field.

## Methods

The neutron dose measurements were performed with a detector system consisting of a PADC (poly(allyl diglycol carbonate)) track etch detector (Thermo Fisher Scientific Inc., Waltham MA, USA) with 2 mm thick radiators (polyethylene and polyethylene with lithium) on both sides. The detectors were provided and read-out by the Division for Radiation Safety and Security of the Paul Scherrer Institut (PSI) [4,5].

**Figure 1:**
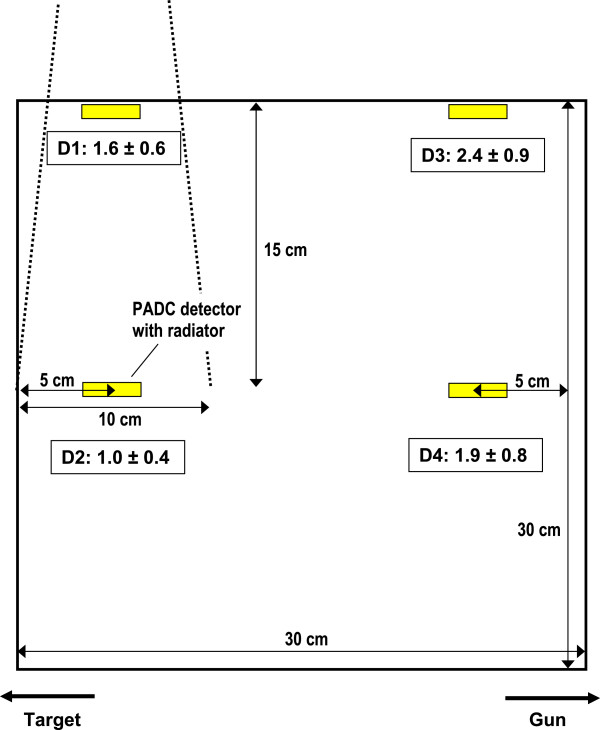
** Experimental set-up.** A 30 x 30 x 30 cm^3^ RW3 solid water cube was irradiated with a 10 x 10 cm^2^ field with a nominal energy of 16 MV at a source surface distance of 85 cm. The radiation beam was placed such that the central ray was 5 cm from the phantom border (isocenter was located at 15 cm depth). The PADC detectors were placed horizontally 5 cm from the edge of the phantom. The numbers in the boxes indicate the ratio of neutron dose equivalent from the IMRT field relative to the open field for the same absorbed dose at position D2.

The detectors were immersed into a 30 x 30 x 30 cm^3^ large RW3 solid water phantom (PTW, Freiburg, Germany). A specifically manufactured holder (acrylic glass) was used to irradiate three detectors simultaneously in order to improve statistics. The experimental setup is depicted in Figure 1. Four measurement positions were chosen: in the radiation field at 0.2 cm depth (D1) as well as in 15 cm depth (D2) and in gun direction outside of the treatment field at 0.2 cm (D3) and 15 cm (D4) depth, respectively. The in-field detectors (D1, D2) were placed on the central ray of the radiation field, the out-of-field detectors (D3, D4) 20 cm away from the central ray.

A linear accelerator (Varian Medical System, Palo Alto CA, USA) was used to produce a photon radiation beam with a nominal energy of 16 MV, having a tissue phantom ratio TPR_20,10_ at 20 cm/10 cm depth of 0.760. Two irradiation techniques have been used. The first field was an open radiation field delivering 75 monitor units (MU) and a total dose of 0.592 Gy in 15 cm depth. The field size of 10 x 10 cm^2^ was defined by the jaws and the multileaf collimator (MLC) was retracted. The second radiation field was intensity-modulated, with a 2.32 cm sweeping MLC gap of 10 cm lateral dimension. With the IMRT field the same dose was delivered as with the open field, but with 225 monitor units. The jaws were positioned to form a 10 x 10 cm^2^ field. The MU-ratio between IMRT and open field was three. Since the sensitivity of the PADC detectors is optimal in a limited dose range, the detectors at the different positions were irradiated with different doses. For the measurement position D1, the radiation field was applied two times, for the positions D2 and D3 six times and for D4 120 times.

The detector output given in tracks per area was first calibrated into personal dose equivalent Hp(10). For this purpose, a subset of the PADC detectors from the measurement batch was mounted on an ISO water slab phantom and irradiated with neutrons from an ^241^Am-Be neutron source. The dose at the detector position was determined with a Berthold LB6411 neutron dose rate meter calibrated at the Physikalisch-Technische Bundesanstalt in Braunschweig, Germany [6], which is used as a secondary standard at PSI. The resulting calibration factor for the used batch of PADC detectors was 16.39 · 10^-3^ mSv cm^2^ ±36% (one standard deviation).

The radiation quality used in the experiment was a photon beam with a nominal energy of 16 MV, which produces neutrons with a different energy distribution than the ^241^Am-Be calibration source. To account for this difference, Monte Carlo simulated neutron spectra in water of a photon beam from a Varian linear accelerator were taken from Kry et al. [7,8]. The detector response normalized to the ^241^Am-Be neutron spectrum from [9] was convolved with the normalized neutron spectra simulated in- and outside of the radiation field resulting in calibration coefficients for the neutron spectra at the four detector positions, which are listed in Table 1. This field calibration procedure is described by Hälg et al. [8]. 

**Table 1:** **Calibration factors for converting tracks per cm**^**2**^**into neutron dose equivalent in mSv for the different positions in the phantom**

**Depth in cm**	**Neutron dose H**_**P**_**(10) (Am-Be)/(tracks per cm**^**2**^**) in mSv cm**^**2**^	**Neutron dose equivalent H (16 MV photons)/H**_**P**_**(10) (Am-Be)**	
		**in-field**	**out-of-field**
0.2	16.39 · 10^-3^	0.91	0.96
15.0	16.39 · 10^-3^	0.71	0.71

The second column represents the initial absolute calibration from tracks per cm^2^ into mSv cm^2^, performed in the ^241^Am-Be neutron field. The third column contains the calibration factors considering the spectral changes with depth.

## Results

The measurement series were performed once for the open field and twice for the IMRT field, leading to three and six measurement values per location, respectively. The average value of the measurements at each location was used to report the results.

The measured neutron dose equivalents at the four locations for the two techniques are listed in Table 2. The ratio of the neutron dose equivalent from the IMRT treatment to the dose resulting from the open field irradiation is shown in the boxes of Figure 1 at the four positions. In the treatment field itself the ratio drops from 1.6 at the surface to 1.0 at 15 cm depth. Outside the treatment field the ratio is 2.4 at the surface and 1.9 at 15 cm depth.

**Table 2:** Neutron dose equivalent in mSv per treatment Gy measured at different depths for an open field and an intensity-modulated field

**Depth in cm**	**Neutron dose equivalent in mSv/Gy for an open field**		**Neutron dose equivalent in mSv/Gy for an IMRT field**	
	**in-field**	**out-of-field**	**in-field**	**out-of-field**
0.2	2.8	1.0	4.5	2.4
15.0	0.20	0.015	0.20	0.029

Dose was prescribed to measurement point D2.

## Discussion

The uncertainty of the calibration and readout of the PADC detectors was estimated by the standard deviation of the readout of two subsets of the detector batch which were used as background detectors or have been irradiated with neutrons from the ^241^Am-Be source for the absolute calibration. It was calculated to be σ = 36%. This was verified by the statistical deviations of the minimum or maximum readout to the mean value of the three repeated measurements at each measurement position, which were in the range of −17% to +30% with an average of less than 10%. The error of the mean was calculated using the Student’s t-distribution ΔD = t · σ/√n = 27% with n = 3 and a corresponding t = 1.32. The total uncertainty was then calculated by error propagation for the ratios of the open and the intensity-modulated field doses. The overall uncertainty of the dose ratios was 39%.

In addition to the detector batch variation of 36% an uncertainty of 17% comes from three sources. One source of around 5% comes from the spectral difference of the calibration conditions (neutrons from an Am-Be source) and the open photon radiotherapy beam. Another source of around 15% comes from the spectral change with depth in the phantom. Both of these uncertainties apply more or less in a similar way to the open and the MLC field and were therefore not considered for the error estimate of the dose ratios. The residual uncertainty of maximal 7% comes from the differences in fluence between the open and the MLC field which was not accounted for, as it is much smaller than the 36% batch uncertainty.

An IMRT treatment delivers more monitor units than a conventional irradiation due to the realization of the intensity modulation. As a consequence, beam-on time is longer for the same delivered dose. Commonly it is assumed that neutron dose equivalent scales with the number of applied monitor units for a radiotherapy treatment irrespectively of the used treatment technique [1,2]. The measurements presented in this report indicate that this may only be true for superficial tissues outside of the treatment field. Deeper lying tissues outside the treatment volume receive around 35% less neutron dose equivalent than expected (three times expected and around two times observed). In the treated volume the dose reduction is even more pronounced with a 45% reduction at the surface and a 65% reduction in larger depth. However, it should be noted here that the irradiated volume is in general much smaller than the body volume which receives only scatter dose. Therefore, if it is assumed that cancer induction is a function of dose and irradiated volume, the neutron dose reduction in the irradiated volume might not have a large impact. In addition the neutron dose equivalent must be viewed always in relation to the primary dose distribution, which is several orders of magnitude larger in the treated volume.

A possible explanation for the measured difference in scaling of the neutron dose with applied monitor units for open and intensity-modulated fields could be the interplay between neutron production and neutron shielding of the multileaf collimator when used in IMRT. Kry et al. [10] found in Monte Carlo simulations of neutron production in a Varian linear accelerator head that the MLC can act as a neutron absorber where the primary photon beam is shielded by the jaws. Zanini et al. [11] did a Monte Carlo study on the photoneutron fields of a Varian linear accelerator operating at a nominal energy of 18 MV. They determined neutron spectra at different locations (inside and outside of the primary field) for different collimation settings. The spectra simulated in that study at 3 cm (as in-field) and 15 cm (as out-of-field) laterally from the isocenter were used to estimate the expected readout of the PADC detectors used in this work. For this purpose the spectra were convolved with the energy dependent detector response function of the PADC detectors determined by PSI [4,5,9]. The absolute neutron fluence per cm^2^ per Gy obtained by Zanini et al. for the static MLC field was multiplied by a factor of three to account for the elongated beam-on time for intensity modulation. The calculated PADC response includes not only the spectral change, but also effects like for example the absorption of neutrons by the MLC. The simulated detector response yielded a neutron dose ratio of 2.4 for the in-field spectrum and 2.0 for the out-of-field spectrum at the surface. These values are in agreement with the measurements of this study. In the work by Zanini et al. it was also shown that for equal field sizes the amount of neutron production in the MLC is smaller than in the jaws.

The neutron spectra simulated in the study by Zanini et al. show a slight shift towards lower energies for the field using the MLC when compared to the open field. This shift lowers also the mean energy, which could be responsible for the change of the ratio between the open field and the intensity-modulated field with depth in the phantom seen in this study, as the moderation of neutrons is energy dependent. Dedicated Monte Carlo simulations would be necessary to assess this in detail.

## Conclusions

The findings of this study indicate that the number of applied monitor units during a radiotherapy treatment alone is not predictive for the neutron dose equivalent the patient receives. Outside the treatment volume in larger depth 35% less neutron dose equivalent was delivered than expected for a MU scaling of a factor of three to deliver the same dose to the target for IMRT compared to the open field. As a consequence, the predicted increase of second cancer induction rates from intensity-modulated treatment techniques at large photon beam energies might have been overestimated. The neutron dose to the patient is dependent on several parameters, such as the type and material of the multileaf collimator and the photon energy, which were not investigated in this work and should be included in further studies.

## Competing interests

The authors declare that they have no competing interests.

## Authors' contributions

RH, JB and US performed the measurements. MB and SM carried out the read-out of the PADC detectors. RH analysed the measured data. US designed the study. All authors read and approved the final manuscript.
